# The interrelationship between dengue incidence and diurnal ranges of temperature and humidity in a Sri Lankan city and its potential applications

**DOI:** 10.3402/gha.v8.29359

**Published:** 2015-12-01

**Authors:** N. D. B. Ehelepola, Kusalika Ariyaratne

**Affiliations:** 1 The Teaching (General) Hospital – Kandy, Kandy, Sri Lanka; 2 Lanka Hydraulic Institute, Moratuwa, Sri Lanka

**Keywords:** dengue, diurnal temperature range, humidity, diurnal range, *Aedes*, Sri Lanka, wavelet analysis, climate change, global warming, neglected diseases

## Abstract

**Background:**

Temperature, humidity, and other weather variables influence dengue transmission. Published studies show how the diurnal fluctuations of temperature around different mean temperatures influence dengue transmission. There are no published studies about the correlation between diurnal range of humidity and dengue transmission.

**Objective:**

The goals of this study were to determine the correlation between dengue incidence and diurnal fluctuations of temperature and humidity in the Sri Lankan city of Kandy and to explore the possibilities of using that information for better control of dengue.

**Design:**

We calculated the weekly dengue incidence in Kandy during the period 2003–2012, after collecting data on all of the reported dengue patients and estimated midyear populations. Data on daily maximum and minimum temperatures and night-time and daytime humidity were obtained from two weather stations, averaged, and converted into weekly data. The number of days per week with a diurnal temperature range (DTR) of >10°C and <10°C and the number of days per week with a diurnal humidity range (DHR) of >20 and <15% were calculated. Wavelet time series analysis was performed to determine the correlation between dengue incidence and diurnal ranges of temperature and humidity.

**Results:**

There were negative correlations between dengue incidence and a DTR >10°C and a DHR >20% with 3.3-week and 4-week lag periods, respectively. Additionally, positive correlations between dengue incidence and a DTR <10°C and a DHR <15% with 3- and 4-week lag periods, respectively, were discovered.

**Conclusions:**

These findings are consistent with the results of previous entomological studies and theoretical models of DTR and dengue transmission correlation. It is important to conduct similar studies on diurnal fluctuations of humidity in the future. We suggest ways and means to use this information for local dengue control and to mitigate the potential effects of the ongoing global reduction of DTR on dengue incidence.

Dengue is a viral infection with severe forms, transmitted by female mosquitoes of the genus *Aedes*. Dengue has been spreading quickly, and the global incidence of dengue has increased 30-fold over the last 50 years (1). The corresponding author, a practicing doctor, observed that the dengue incidence of the city of Kandy tends to be lower in the February–April period, even during years with epidemics, and decided to further probe into this phenomenon. He also noticed that during the first 3 months of the year diurnal fluctuations of temperature and humidity were high in Kandy, and he suspected a possible correlation between those fluctuations and the low dengue incidence.

Here, *diurnal temperature range* (DTR) refers to the difference between daily maximum and minimum temperatures, whereas *diurnal humidity range* (DHR) indicates the difference between daily night-time and daytime relative humidity.

Pioneering studies on dengue–DTR correlation were published after noticing that a large DTR was followed by a decline in dengue incidence in Mae Sot, Thailand. There have been three such noteworthy studies. One international team conducted detailed laboratory experiments and modelling (2) and reported that vector mosquitoes were less susceptible to virus infection (reduced vector competence) and died faster under larger DTR around the same mean temperature. Vectorial capacity is influenced linearly by variations in vector competence and exponentially by variations in survival (2). Their thermodynamic model predicted at mean temperatures >18°C (such as in Kandy) that larger DTR would reduce dengue transmission.

The effects of the DTR of Mae Sot on immature forms of *Aedes aegypti* were studied in the laboratory (3) by another team. They found that a large DTR extended immature development time, lowered larval survival, and reduced adult female reproductive output relative to a constant 26°C temperature or a small DTR. *Aedes aegypti* vector competence was assessed under DTR conditions more realistically mimicking those of Mae Sot by a third team. They found that a large DTR around 26°C meant reduced midgut infection rates and tended to increase the dengue virus's extrinsic incubation period (4). All of this evidence shows that large DTR is unfavourable for dengue transmission.

There is a global trend for DTR to decline with ongoing climate changes. Recently, a different team estimated future global dengue epidemic potential trends based on the effects of temperature and DTR on the vectorial capacity of *A. aegypti* (5). We were not able to find even a single published study about the correlation between DHR and dengue transmission, although many past researchers have studied the correlation of dengue with humidity.

Many weather variables other than DTR have been shown to affect dengue transmission via impact on the life cycle of the vector and therefore the part of the life cycle of the dengue virus inside the vector (6, 7). These variables include rainfall in millimetres, number of rainy (and wet) days during a certain period, daytime and night-time, as well as average humidity, minimum, mean, and maximum temperature, hours of sunshine, wind velocity, wind run (wind run = wind velocity×duration), and atmospheric pressure (6–11). All of these variables except atmospheric pressure have been demonstrated to be correlated with the dengue incidence of Kandy (7). Ehelepola et al. (7) present summaries of recent dengue weather correlation studies done in Kandy, Sri Lanka, and other South Asian countries. The El Niño southern oscillation is also known to influence dengue transmission in certain localities (6, 8, 10).

## Study setting

Kandy is located at 7°17′47″ N, 80°38′6″ E, and 500 m above sea level. It is the largest city in the central highlands of Sri Lanka. The study area was 28.53 km^2^. The estimated midterm resident population for this study period was 114,600. During the study period, for the city of Kandy, the average mean daily temperature was estimated at 25.1°C, and the averages of minimum and maximum temperatures, respectively, were 20.8°C and 29.6°C. The average daytime and night-time relative humidity were estimated to be 73 and 88%, respectively, for the same period. In Sri Lanka, the daily temperature fluctuations are greater than the annual fluctuations.

## Objectives and hypotheses

The first objective was to determine the correlation between local dengue incidence and DTR. The second objective was to investigate the correlation between dengue incidence and DHR. We then explored possible ways to improve dengue control using those correlation patterns and other available evidence.

Our hypothesis was that large diurnal fluctuations of both temperature and humidity have a negative correlation with the dengue incidence in the city of Kandy.

## Methods

### Ethical statement

The numbers of notified dengue patients were collected without any information about their identity. We used the data from a previous study after obtaining written consent from our co-authors for that study. We obtained clearance from the ethical review committee of the Peradeniya medical faculty (2012/EC/75).

### Data

The number of all notified dengue patients of the city of Kandy from 1 January 2003, to 31 December 2012 was collected from the registers at the office of the Medical Officer of Health of the city. We obtained estimated midyear population data of the city for the same period from the Kandy divisional secretariat office. Daily minimum and maximum temperatures and night-time and daytime humidity data pertaining to the same period were acquired from the Katugastota and Gannoruwa weather stations. We decided to take the average values of two weather stations, as there are small differences in weather in the northern and southern sides of the city, and there are many people who live on one side and work on the other side of the city.

### Analysis

We calculated the weekly dengue incidence of Kandy for 2003–2012. Weather data were converted to weekly values and averaged, and then weekly averages of the diurnal ranges of temperature and humidity were calculated.

**Fig. 1:**
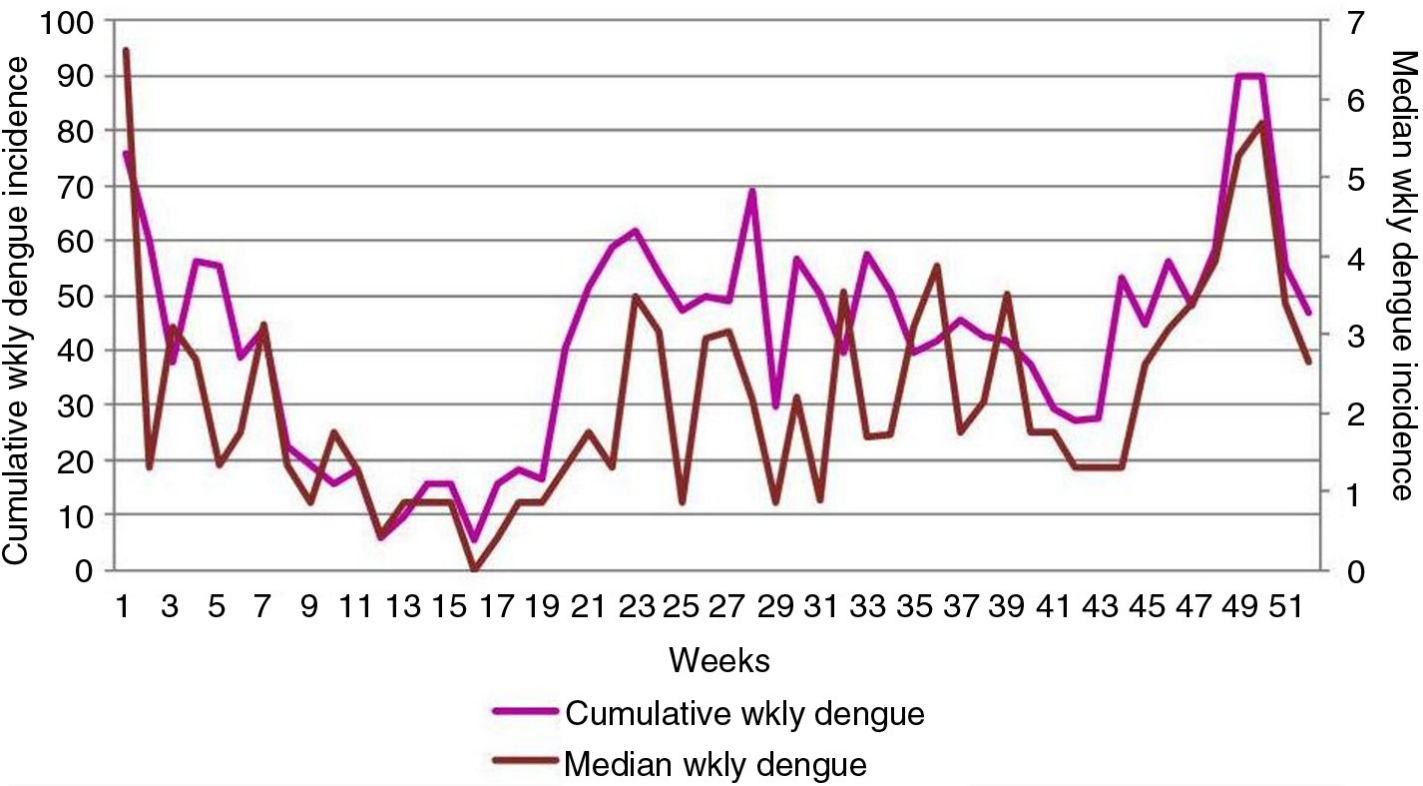
Changes in the cumulative weekly dengue incidence (per 100,000 population) and median of weekly dengue incidences (per 100,000 population) during the course of the year, for 2003–2012. x-Axis: weeks; primary y-axis: cumulative weekly dengue incidence; secondary y-axis: median of weekly dengue incidences.

**Fig. 2:**
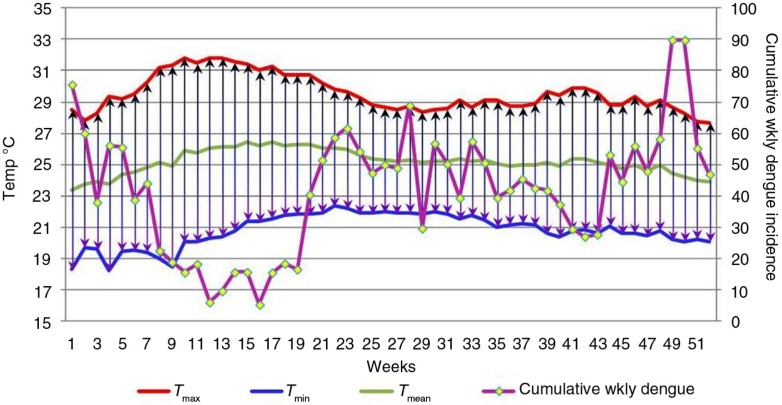
Changes in weekly minimum (*T*_min_), maximum (*T*_max_), and mean (*T*_mean_) temperatures and cumulative weekly dengue incidence over the course of all 52 weeks of the year, for 2003–2012. x-Axis: weeks; primary y-axis: temperature in degrees Celsius; secondary y-axis: cumulative weekly dengue incidence.

**Fig. 3:**
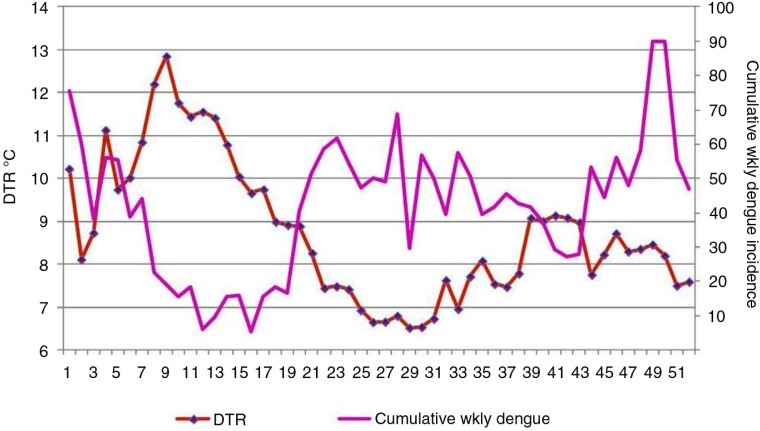
Changes in weekly diurnal temperature range (DTR) in °C (red) and cumulative weekly dengue incidence (purple) over the course of all 52 weeks of the year, 2002–2012. x-Axis: weeks; primary y-axis: DTR in degrees Celsius; secondary y-axis: cumulative weekly dengue incidence.

**Fig. 4:**
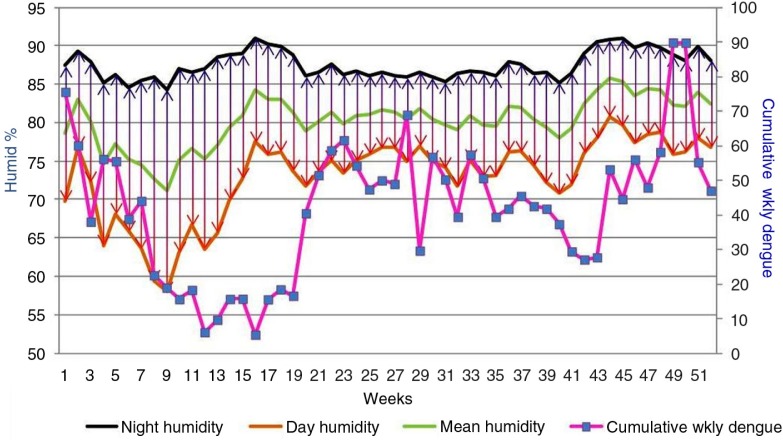
Changes in weekly night-time humidity, daytime humidity, and cumulative weekly dengue incidence over the course of all 52 weeks of the year for the period 2003–2012. x-Axis: weeks; primary y-axis: relative humidity (%); secondary y-axis: cumulative weekly dengue incidence.

**Fig. 5:**
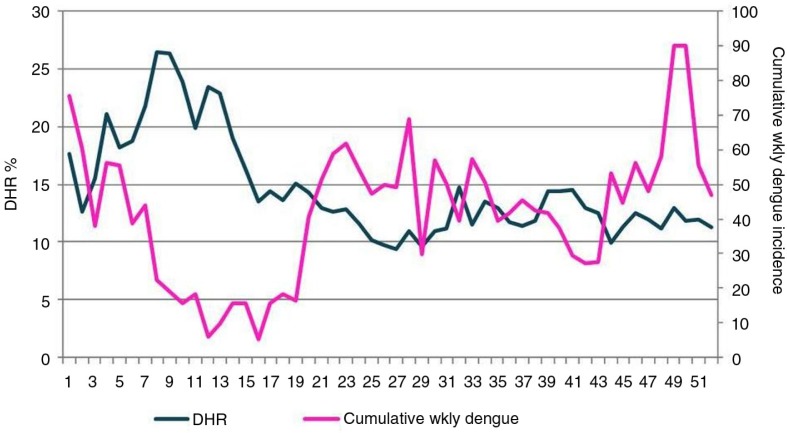
Changes in weekly diurnal humidity range % (DHR) and cumulative weekly dengue incidence over the course of all 52 weeks of the year, 2003–2012. x-Axis: weeks; primary y-axis: DHR in percent; secondary y-axis: cumulative weekly dengue incidence.

We plotted graphs of the total (cumulative)weekly dengue incidence versus weekly averages of the diurnal ranges of temperature and humidity over the course of 52 weeks of the year and observed the correlation patterns for the period of 2003–2012. Peaks of DTR and DHR were followed by troughs of dengue incidence after lag periods of a few weeks. Considering the patterns observed in Figs. 1–5, we decided to study these patterns in depth using wavelet analysis. Over the course of a week, there can be a few days with extreme DTR or DHR values. We performed wavelet analysis to find the relationship between the number (count) of days per week where DTR is >10°C and <10°C and the number of days in which each week's DHR is >20 and <15%, as well as the dengue incidence.

### Details of wavelet analysis

Dengue weather correlation is non-stationary, and wavelet analysis is very suitable to detect such correlations. In wavelet analysis, a suitable window is selected, this window is shifted along the signal, and for every position the spectrum is calculated. This process is then repeated many times with a slightly shorter and longer window for every new cycle. With wavelet transform, the result will be a collection of time-frequency representations of the signal with different resolutions. The importance of wavelet transform lies in the fact that it can be used to analyse time series that contain non-stationary power at many different frequencies. By decomposing a time series into time-frequency space, it is possible to very easily determine both the dominant modes of variability and how those modes vary in time. Cross wavelet transform (XWT) and wavelet coherence (WTC) can be used for examining relationships in time-frequency space between two time series. Whereas continuous wavelet transform (CWT) is a common tool for analysing localised intermittent oscillations in a time series, it is very often desirable to examine two time series together that may be expected to be linked in some way.

The CWT expands the time series into time-frequency space. The CWT decomposes the time series into time-frequency space, enabling the identification of both the dominant modes of variability and how those modes vary with time.

The idea behind the CWT is to apply the wavelet as a band-pass filter to the time series. The wavelet is stretched in time by varying its scale, *s*, so that *η*=*st*, and normalising it to have unit energy.

The CWT of a time series *X*_*n*_, *n*=1, 2, …, *N*, with uniform time step *δt* is defined as the convolution of *X*_*n*_ with the scaled and normalised wavelet (12).

$$W_{n}^{X}(s)=\sqrt{\frac{\delta t}{s}}\sum_{n^{\prime }=1}^{N}X_{n^{\prime }}\psi_{0}\left[\left(n^{\prime }-n\right)\frac{\delta t}{s}\right]$$

Wavelet power is calculated as follows (12):

$${|W_{n}^{X}(s)|}^{2}$$

**Fig. 6:**
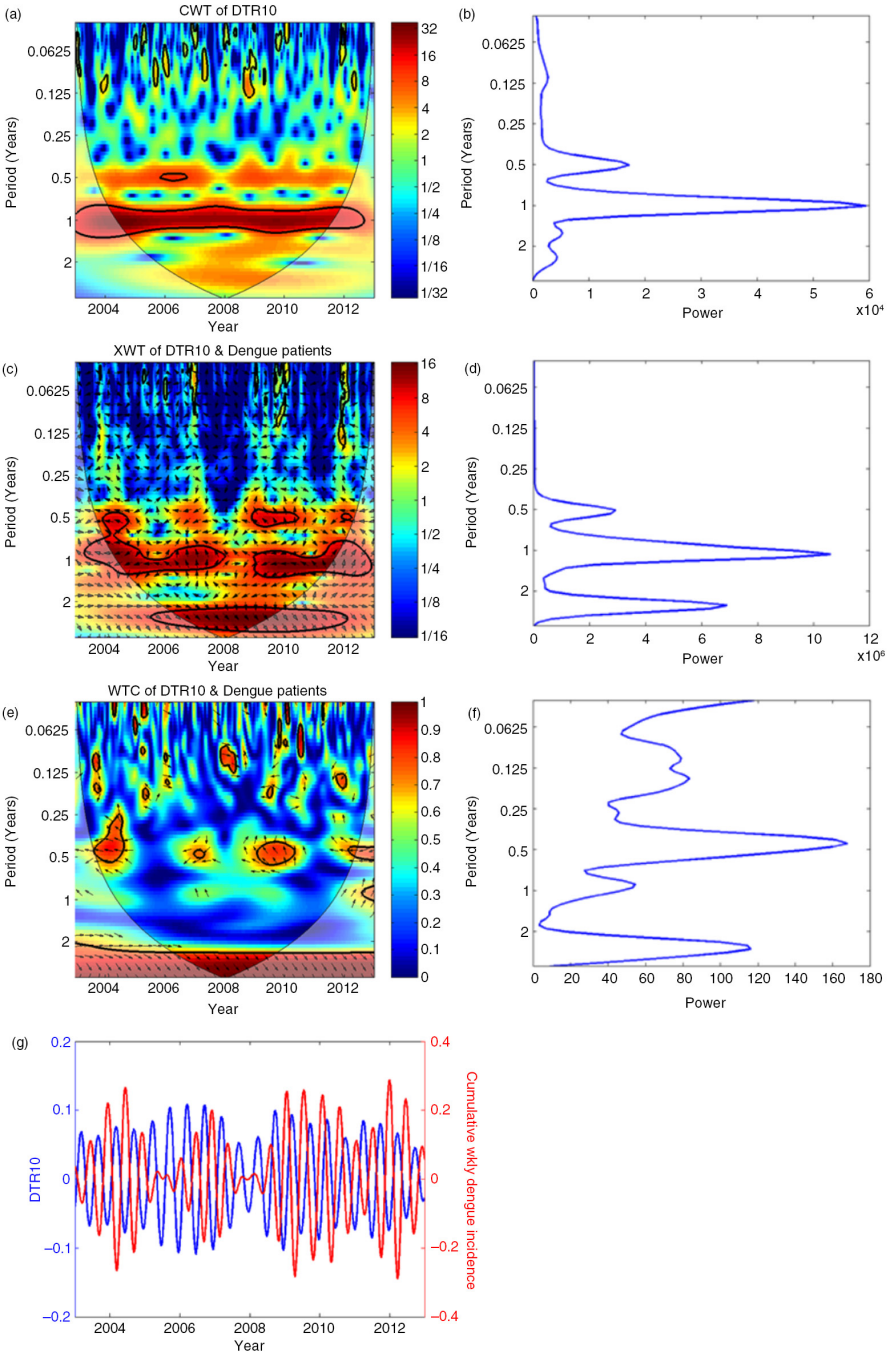
Results of the wavelet analysis of weekly dengue incidence versus number of days per week with DTR >10°C as a sample of wavelet analysis results. (a) Continuous wavelet transform (CWT) variations; (b) wavelet power of CWT; (c) cross wavelet transform (XWT) variations; (d) wavelet power of XWT; (e) wavelet coherence (WTC); (f) wavelet power of WTC; (g) reconstructed time series for selected periods. In Fig. a, c, and e, there are colour-coded columns on the right side of the main figure. Those indicate the strength of coherence in which dark blue and dark red indicate the lowest and highest coherence, respectively. Areas inside the thin parabolic black line (cone of influence) are not influenced by the edges of data. Term period in the vertical axis indicates duration of cycle (in years).

Figure 6 shows DTR >10°C wavelet transform variations. Figure 6a shows the CWT variation plot as a contour map, and Fig. 6b shows the variation of wavelet power with period.

In order to check the possibility of common power, the XWT was carried out, and the variations are shown in Fig. 6c and d.

The XWT finds regions in time-frequency space where the time series show high common power.

The XWT of two time series *X*_*n*_ and *Y*_*n*_ is defined as in Ref. (12):

$$Y*W^{XY}=W^{X}W$$

where * denotes complex conjugation.

Cross wavelet power (12) is defined as |*W^XY^*|

Figure 6c shows the XWT variation plot as a contour map, and Fig. 6d shows the variation of wavelet power with period.

In order to check the possibility of having a causality effect, the WTC was calculated, and the variations are shown in Fig. 6e and f.

*Wavelet coherence* is defined as the square of the cross spectrum normalised by the individual power spectra. This gives a quantity between 0 and 1 and measures the cross correlation between two time series as a function of frequency.

WTC finds regions in time-frequency space where the two time series co-vary, but do not necessarily have high power. Coherence is a measure of the intensity of the covariance of the two series in time-frequency space, unlike the cross wavelet power, which is a measure of the common power. It was the aim of this study to examine two time series together that may be expected to be linked in some way. If there are regions in time-frequency space with large common power that have a consistent phase relationship, it suggests causality between time series (12).

*WTC* (12) is defined as follows:

$$R_{n}^{2}(s)=\frac{{|S\left(s^{-1}W_{n}^{XY}(s)\right)|}^{2}}{S\left(s^{-1}{|W_{n}^{X}(s)|}^{2}\right).S\left(s^{-1}{|W_{n}^{Y}(s)|}^{2}\right)}$$

where *S* is a smoothing operator.

Note that this definition closely resembles that of a traditional correlation coefficient, and it is useful to think of the WTC as a localised correlation coefficient in time-frequency space.

Figure 6e shows the WTC variation plot as a contour map, and Fig. 6f shows the variation of wavelet power with period.

In order to find the lag times, the time series were reconstructed for the period that gave the maximum power. Figure 6g shows the reconstructed time series.

Since the wavelet transform is a band-pass filter with a known wavelet function, it is possible to reconstruct the original time series.

The reconstructed time series is represented as follows:

$$x_{n}=\frac{\begin{array}{c} s\\ \left(j\right)\\ W_{n}\\ R\\ \delta j\delta t^{\frac{1}{2}} \end{array}}{C_{\delta }\psi_{0}(0)}\sum_{J=0}^{J}$$

where *ψ*_0_(0) removes the energy scaling, while $s_{j}^{\frac{1}{2}}$ converts the wavelet transform to an energy density. The factor *C*_*δ*_ is a constant for each wavelet function.

Considering the above equation and by summing over a subset of the scales, it is possible to construct a wavelet-filtered time series. In the present study, the period that gave the highest coherence among the dengue cases and each of the weather parameters was identified. The wavelet-filtered time series for this period were reconstructed, and the leading/lag times were estimated.

Refer to Fig. 6g for reconstructed time series. Both variations of dengue incidence and weather parameter DTR were plotted together for easy comparison.

MATLAB R2013a software (MathWorks Inc., Natick, MA, USA) was used for wavelet analysis, and Microsoft Office 2007 software was used for other work.

## Results

Figure 1 shows the changes in cumulative weekly dengue incidence and median of weekly dengue incidences over the course of all 52 weeks of the year, for the time span 2003–2012. We considered the total number of dengue incidences of the first week of all 10 years as the cumulative dengue incidence for the first week, and the same method was used for other weeks.

The decline of dengue incidence in February through late April is obvious in Fig. 1.

Figure 2 shows the changes in weekly minimum, maximum, and mean temperatures and cumulative weekly dengue incidence over the course of all 52 weeks of the year, for the time span 2003–2012. The decline of dengue a few weeks after weeks with a large DTR is apparent here.

Figure 3 illustrates the changes in weekly DTR and cumulative weekly dengue incidence over the course of the year, for the period 2003–2012. The decline of dengue a few weeks following a DTR >10°C and the rise of dengue following a DTR <10°C is shown here.

Figures 2 and 3 show the rise of DTR during the initial weeks of the year, and it reaches its peak in mid-February, followed by a reduction in dengue incidence after a lag period of a few weeks. A similar phenomenon occurs in October with a lesser magnitude.

Figure 4 illustrates the changes in weekly night-time humidity, daytime humidity, and cumulative weekly dengue incidence over the course of all 52 weeks of the year for 2003–2012. The decline in dengue a few weeks following large fluctuations of the DHR is depicted here.

Figure 5 shows the changes in weekly DHR and cumulative weekly dengue incidence for the same period. We can see the decline in dengue following a DHR >20% and the rise of dengue following a small (<15%) DHR.

Figures 4 and 5 show the rise of diurnal fluctuation in humidity, which peaks in mid-February and is followed by the decline of dengue incidence with a couple of weeks’ lag. A similar phenomenon of lesser magnitude happens in October.

In Kandy, during January and February, the night-time (minimum) temperature is low and the daytime is hotter compared with the other months. Maximum and mean temperatures that were low during December gradually rise until mid-March. Large diurnal humidity fluctuations from February to March occur mainly due to low daytime humidity during this period.

Wavelet analysis results of weekly dengue incidence versus number of days per week with a DTR >10°C are presented here as a sample of results of the wavelet analysis.

Wavelet analysis results showed that the number of days per week that DTR was >10°C was negatively correlated with the weekly dengue incidence, with a lag period of 3.3 weeks. The number of days per week with DTR <10°C versus weekly dengue incidence showed a positive correlation, with an average 3-week lag. However, this correlation pattern was not seen in some months (in 2006–2007 and 2012). Similarly, we observed a positive correlation with a 3.8-week lag only during 2008–2012, when we studied the number of days per week with DTR <8°C versus weekly dengue incidence. We shall not forget that dengue correlation with weather is non-stationary.

Number of days per week with DHR >20% was negatively correlated with the weekly dengue incidence, and number of days per week with DHR <15% was positively correlated with dengue incidence; both had a 4-week lag.

However, these cut-off points that we determined by observing Figs. 3–5 are not intended to be precise thresholds. To calculate precise threshold values, more analyses have to be done. At the present level of analysis, it is difficult to interpret the dengue transmission pattern of 15–20% DHR range. Nevertheless, that did not deter us from pursuing our objectives.

## Discussion

Results of the analysis confirmed our hypothesis that large diurnal fluctuations in temperature (which occur in January–April) and humidity (which occur in February–mid-March) are negatively correlated with the dengue incidence of Kandy that occurs with a lag period of 3.3–4 weeks. A similar phenomenon with lesser magnitude occurs in mid-September–October. Dengue incidence in Kandy is high from late October through February of the following year.

Weather and many other factors such as the introduction of new virus phenotypes to the population, herd immunity of the population, effects of preventive measures, population movements, housing and garbage disposal, and attitudes of people also affect local dengue incidence (7).

Once a healthy person gets bitten by a vector with dengue virus in its saliva, it takes approximately 2–3 weeks for that person to show symptoms, visit a hospital, get diagnosed, and then get entered into the registers from which we derived these data. These results show that DTR and DHR affect dengue incidence with 3–4 weeks of lag. Hence, both DTR and DHR seem to affect dengue transmission quickly, within days. Likely mechanisms are a combination of poor penetration of the mosquito gut mucosa by the dengue virus (2), an increase in extrinsic incubation period (4), and dengue virus-infected vectors dying faster (2) due to large DTR.

We failed to find any published information about the possible mechanism(s) to explain the effect of large DHR on the vector and dengue virus. During the first 3 months of the year, Kandy gets less rain. It is well known that humidity is high during rainy days and low during dry days. We cannot exclude the possibility of low rainfall and high DTR, but not large DHR, causing a decline of dengue during that period without doing further studies. Like temperature, humidity has also been shown to affect *Aedes* reproduction, longevity, and feeding (6, 7). We believe that the best possible way to verify dengue correlation with DHR is to conduct studies similar to the investigations that found effects of large DTR on the vector and dengue virus (2–4). We anticipate carrying out such a study in the future.

### Comparison with the results of related studies

From mid-March to mid-April in Kandy, there is an average DTR >10°C around the mean temperature of 26°C, which is similar to Mae Sot, the location where DTR was thoroughly studied in the past (2, 4). However, Mae Sot is situated at a higher northern latitude and there is a much larger landmass surrounding it. Hence, understandably it has more extreme DTR fluctuations. In Kandy, a relatively smaller DTR is also negatively associated with local dengue incidence; a DTR >10°C decreases dengue, while a DTR <10°C is conducive to dengue transmission. It is very interesting to see that our results derived from epidemiological data agree with existing mathematical models of the effects of temperature on dengue transmission (2, 5), especially the relative vectorial capacity (rVc) of *Aedes* as depicted in Fig. 1 and Supplementary Fig. 1 of Ref. (5). A DTR approximately 8°C around the mean temperature of 26°C was shown to be slightly favourable or have no effect on dengue transmission by one entomological study (4) done in a laboratory. Our Fig. 3 shows that a DTR of approximately 8°C is conducive for dengue transmission. The count of days per week with DTR <8°C versus weekly dengue incidence wavelet analysis also showed a positive correlation with a 3.8-week lag, but this correlation was not seen during the full time period; it was apparent only during 2008–2012. It is interesting to see that our results agree with previous laboratory study results, as well.

In many parts of the world, DTR is becoming smaller with climate changes (13), and in fact the DTR is described as an index of global climate changes. The results of the present study indicate that small changes in DTR (even <2°C) from a 10°C baseline can cause dramatic changes in the dengue transmission patterns, at least in some localities. Such changes may happen in many locations in the coming centuries. A mathematical modelling of rVc regarding this phenomenon already exists (5), and more epidemiological studies on this topic would be useful to confirm the usefulness of that model and for further refinements. According to that model (Fig. 1 of Ref. 5), if Kandy had a mean temperature <21°C or >33°C, a large DTR would have increased and a small DTR would have reduced dengue incidence if dengue incidence still followed rVc.

There are other differences between Mae Sot and Kandy that can be identified as follows. The city of Kandy is more urban, with a much higher population density. In Kandy, the most abundant dengue vector is *Aedes albopictus* (not *A. aegypti*). No information is available on the proportions of the contribution by each of these two vectors to the local dengue incidence. All four serotypes of dengue virus co-circulate in Sri Lanka. The published studies on dengue versus DTR correlations were performed by considering *A. aegypti* of Thai origin as the vector (2–4), using one serotype of dengue virus, and for a DTR similar to that of Mae Sot. It is interesting to note that in Kandy a similar pattern of correlation also occurs between the DTR and local dengue incidence in spite of the differences with Mae Sot. Mathematical model graphs of how rVc changes with the mean temperature and DTR that are created by considering only *A. aegypti* as the dengue vector (5) are in agreement with the results we obtained for Kandy city, where *A. albopictus* is the most abundant dengue vector. This agreement indicates that the rVc for both of the above dengue vectors are likely to have similar relationships with temperature. This finding is remarkable, as we can apply those existing graphs to vast areas where both dengue vectors are present, to forecast the potential rise of dengue with the ongoing climate changes. However, we believe that further studies are needed to confirm this.

After completion of this study, the authors noted a very recent study conducted in Dhaka, Bangladesh, over a 10-year period (14). Dhaka is situated at a higher latitude and lower elevation and has a higher population density compared with Kandy. It also has an average mean temperature of approximately 25°C and annual rainfall of about 2,000 millimetres, as in Kandy. Interestingly, they found that interaction between mean temperature and DTR significantly influenced dengue transmission with a lag of 4 weeks, and that high mean temperature with low DTR increased dengue incidence. Dengue incidence in Kandy was also positively correlated with maximum, mean, and minimum temperatures (7). We believe that the results of the present study will contribute to a better understanding of dengue correlation with weather, especially dengue–DTR correlation, and that our results confirm the value of those previous findings.

The first published study (2011) on dengue–DTR correlation examined the effect of DTR after noting that seasonal fluctuations in dengue transmission are likely to be governed by factors other than vector abundance in the Mae Sot area (2). According to the researchers, immature forms of *A. aegypti* in Thailand develop principally in managed water containers; hence, the rainfall does not strongly affect dengue transmission. Dengue incidence in the city of Kandy is positively correlated with rainfall in millimetres and the number of rainy days, as well as wet days (7). Another study done in the Kandy area showed that the most positive vector breeding habitats were outdoors (15), and thus they were affected by rain. Interestingly, in spite of the correlation with rain, dengue incidence in Kandy is correlated with both DTR and DHR, as well. Rainfall in millimetres is lower and rainy days are fewer in Kandy during the first 3 months of the year, and a similar rainfall pattern occurs in Mae Sot, as well (16).

A past study showed low *Aedes* egg/ovitrap counts and low larval density indices during the first 3 months of the year in the Kandy area (15), which indicates lower vector density. Large DTR also reduced the vector mosquito population in laboratory studies (2, 3). Therefore, reduction of the vector population is likely to be a result caused by both low rainfall and high DTR during this period.

### Potential applications for local dengue control

A certain concentration of vector mosquitoes is needed to cause a dengue epidemic. Mosquito larval indices are used as proxies to indicate vector concentration. In the Kandy area, these indices are high (15). However, during the first 3 months of the year, these indices are relatively low (15). In Sri Lanka, including Kandy, vector population control is the backbone of dengue control. Control measures are vigorous, usually after the onset of the rainy season and when dengue incidence rises. If we take advantage of weather that is unfavourable for dengue transmission (high DTR and DHR and low rainfall) and continue dengue control programs vigorously in the first 3 months, we may be able to control vector population and dengue transmission to a level that makes initiation of an epidemic in the approaching several weeks unlikely. This discovery is important considering that the 2004 and 2009 dengue epidemics had started by May in Kandy. Postponing or preventing the onset of such epidemics will help to reduce dengue morbidity and mortality.

### Potential for rise of dengue with declining DTR due to global climate changes: a proposal to mitigate that problem and additional benefits of that proposal

Ongoing climate changes are among the greatest challenges to human civilisation. Global warming has been attributed to a larger increase in the night-time (minimum) temperature than the daytime (maximum) temperature (13). In other words, global warming tends to reduce DTR while increasing both minimum and maximum temperatures. Published studies have indicated that the warming trend has become faster over the period 1961–2010 in Sri Lanka, with an increase of 0.01–0.03°C per year, depending on the location (17). Another study showed accelerated warming in the 1960–2000 period compared with the 1869–1960 period in Sri Lanka (18). In Kandy (in contrast to the global trend), the maximum temperature has increased more than the minimum temperature, although both increased during 1961–2001 (18).

Considering all of the above, we can expect a rise of dengue in many parts of Sri Lanka and in the world due to the rise of mean temperature and lower DTR due to global warming.

Within an 18–29°C mean temperature range, the rise in mean temperature and reduction of DTR will help to produce more *Aedes* vectors with dengue virus in their saliva. Higher temperatures increase their biting frequency, as well, putting an enormous population (more than one billion people in South Asia alone) at a higher risk of dengue.

The authors believe that it is very important to extrapolate to what we can do to mitigate this risk. Due to the absence of any published proposals, we suggest a simple, affordable, practical solution. Our suggestion is to popularise the routine application of topical insect repellents in high dengue risk localities, especially at dawn and dusk, as *Aedes* is known to bite mostly during these times. Encouragement of the routine use of topical mosquito repellents by patients in high-risk areas with pyrexia, headache, arthralgia, and myalgia until dengue is excluded will be useful to reduce dengue virus transmission (7). Hospital-acquired dengue is common in Kandy and in Sri Lanka in spite of provision of mosquito nets to hospitalised dengue patients. Application of topical mosquito repellents to all hospitalised dengue patients, particularly in months with low DTR, will help to control this problem. Considering that the reduction in DTR also favours *Aedes* population growth, these recommendations are intended to supplement (rather than replace) existing dengue control measures aimed at reduction of the vector population. Not only synthetic repellents like DEET but also locally available natural products like citronella oil and margosa oil obtained from plants grown in tropical countries will be useful and will be more acceptable to certain people and communities.

We think that it is worthwhile to mention the additional benefits of these recommendations, even though they are slightly separate from the main topic. The current method in Sri Lanka to counter adult vectors with dengue virus in their saliva is fogging, which is mainly employed to kill such vectors around the homes of reported dengue patients. It is effective for that task. However, it is costly, affected by weather, and both the insecticides (malathion) and kerosene (or diesel oil) added to make the fog have adverse impacts on both the health of people and the environment due to acute and chronic exposure. Therefore, it is unsuitable for wider and more frequent use to counter the effects of rising temperature and declining DTR. By using water-based insecticides, the adverse effects of petroleum products can be avoided. Mosquitoes develop resistance to insecticides. To millions who seriously practice Buddhism and Jainism, killing any animal, including mosquitoes, is unacceptable. The use of fogging to kill adult *Aedes* also kills non-bloodsucking mosquitoes such as *Toxorhynchites* and other insects like dragonflies; the larvae of both feed on *Aedes* and other useful insects like bees. Topical repellents do not entail such problems; they only cause rare allergies. As individuals take responsibility for preventing their own infection, the ‘I am doing this for my own safety’ mindset may provide additional motivation for people to implement the application of mosquito repellents. On the other hand, this method will be poorly implemented in an unmotivated population. We think that pilot studies will be helpful to determine the efficacy of these proposals.

### Limitations of the study

The accuracy of the results of this study depended on the accuracy of the data related to weather, population, and dengue incidence. Reported dengue cases are only a fraction of the total dengue cases, but they were the best practical option available. ‘Urban heat islands’ were reported in Kandy, in localities without vegetation. In those areas, temperatures can rise up to 2°C more than in a comparable green area during the daytime, and diurnal fluctuation in humidity is also different in those areas (19). However, a larger percentage of the city area is green. Taking weather data from two weather stations and averaging may have compensated for weather data differences within the city area.

## Conclusions

Dengue incidence in the city of Kandy is negatively correlated with large diurnal fluctuations of both temperature and humidity. We propose taking advantage of these weather conditions and augment dengue preventive activities during the first 3 months of the year to minimise dengue transmission and vector population.

To mitigate the rising risk of dengue due to rising global temperatures with falling DTR, promotion of the use of topical mosquito repellents will be useful. Further studies to determine how a large DHR reduces dengue transmission deserve serious attention.
